# Nonverbal synchrony in subjects with hearing impairment and their significant others

**DOI:** 10.3389/fpsyg.2022.964547

**Published:** 2022-08-18

**Authors:** Christiane Völter, Kirsten Oberländer, Sophie Mertens, Fabian T. Ramseyer

**Affiliations:** ^1^Department of Otorhinolaryngology, Head and Neck Surgery, Catholic Hospital Bochum, Bochum, Germany; ^2^Clinical Psychology and Psychotherapy, University of Bern, Bern, Switzerland

**Keywords:** hearing loss, nonverbal synchrony, interpersonal relations, dyadic interaction, auditory rehabilitation

## Abstract

**Introduction:**

Hearing loss has a great impact on the people affected, their close partner and the interaction between both, as oral communication is restricted. Nonverbal communication, which expresses emotions and includes implicit information on interpersonal relationship, has rarely been studied in people with hearing impairment (PHI). In psychological settings, non-verbal synchrony of body movements in dyads is a reliable method to study interpersonal relationship.

**Material and methods:**

A 10-min social interaction was videorecorded in 39 PHI (29 spouses and 10 parent-child dyads) and their significant others (SOs). Nonverbal synchrony, which means the nonverbal behaviors of two interacting persons (referring to both general synchrony and the role of leading) and verbal interaction (percentage of speech, frequency of repetitions, and queries) were analyzed by computer algorithms and observer ratings. Hearing-related quality of life, coping mechanisms, general psychopathology, quality of relationship, and burden of hearing loss experienced by SOs were assessed using questionnaires.

**Results:**

In the 39 dyads, true nonverbal synchrony differed from pseudosynchrony [*t*_(43.4)_ = 2.41; *p* = 0.02] with a medium effect size (*d* = 0.42). Gender of PHI had a significant effect on general synchrony (*p* = 0.025) and on leading by SOs (*p* = 0.017). Age gap correlated with synchronic movements (*p* = 0.047). Very short duration of hearing impairment was associated with lower nonverbal synchrony in the role of leading by SOs (*p* = 0.031). Feeling of closeness by PHI correlated negatively with the role of leading by SOs (*p* > 0.001) and feeling of closeness by SOs was positively associated with leading by PHI (*p* = 0.015). No correlation was detected between nonverbal synchrony and other questionnaires. Burden experienced by the SOs was higher in SOs who reported less closeness (*p* = 0.014).

**Discussion:**

A longer hearing impairment leads to more nonverbal leading by SOs compared to PHI with very short duration of hearing loss, possibly because of the long-lasting imbalance in communication. If PHI felt more closeness, SOs led less and vice versa. Burden experienced by SOs negatively correlated with closeness reported by SOs. Use of nonverbal signals and communication might help to improve benefits of auditory rehabilitation for PHI and decrease burden experienced by SOs.

## Introduction

Hearing impairment is the third most common chronic disease and has numerous effects (Scarinci et al., 2008; Chen et al., 2016; Lawrence et al., 2020) on physical, cognitive, mental, and social health causing a decrease in quality of life (QoL) in up to 49.1% of the people affected (Dalton et al., 2003; Andries et al., 2020; Lawrence et al., 2020; Bott and Saunders, 2021).

Effective communication, which is crucial for social interaction, is highly complicated in people with hearing impairment (PHI), and may lead to frustration and resentment, potentially affecting interaction quality in these individuals (Greef, 2000). Problems are numerous and include misunderstanding in communication in general, and—more specifically—changes in the frequency, or type of communication, or the inability to repair a breakdown of communication. Conversational exchanges are often hindered by a loss of spontaneity and the difficulty to share small unexpected observations in everyday interactions, which has a strong impact on conjugal relationships, as sharing is a basic element in conjugal relatedness (Weiss and Heyman, 2004).

So far, hearing rehabilitation has mainly focused on PHI. Recently, the role of significant others (SOs) has increasingly come into focus (Ask et al., 2010; Ekberg et al., 2021; Scarinci et al., 2021). Communication self-efficacy training may lead to improved communication abilities of both PHI and SOs (Roberts and Delich, 2020; Delich and Roberts, 2021). However, as explained in more detail in the concept of the International Classification of Functioning and Diseases (ICF), PHI and their close partners experience and manage hearing loss in the context of their relationship, and SOs also suffer from the hearing impairment of the partner, as indicated by the term “third-party disability” [Word Health Organization (WHO), 2001; Vas et al., 2017]. Three specific risk factors are associated with severe third-party disability, (1) relationship satisfaction, (2) spousal age difference, and (3) spouses' perception of their partners' hearing disability (Scarinci et al., 2012). Coping strategies, which dyads use, seem to be directly related with QoL of the PHI and their partners. Targeted interventions are proposed to help PHI and their partners to implement more effective coping strategies (Blazer and Tucci, 2019; Lazzarotto et al., 2019).

People with hearing impairment need to be considered as interconnected social beings, who are continually and reciprocally engaged in social interactions, which may show signs of embodied cognition such as interpersonal synchronization (Tschacher and Bergomi, 2011). Interpersonal synchrony is fundamental to human beings and occurs in various areas from the early beginning between mother and child (Feldman, 2007; Reyna and Pickler, 2009; Tschacher et al., 2012; Bell, 2020; Koole et al., 2020; Levy et al., 2021). It appears to constitute social connection and understanding (Rennung and Göritz, 2016). These processes are essential for our navigation in the social world; one of the beneficial effects is their function as a kind of “social glue” (Lakin et al., 2003) that strengthens the connection between people both in everyday life (Ayache et al., 2021; Cheng et al., 2022) and in professional settings, such as psychotherapy (Wiltshire et al., 2020), medical settings (Hamel et al., 2022), or in experiments and student interactions (Mogan et al., 2017).

The coordination of nonverbal behavior between interacting individuals usually occurs in the absence of conscious control and has first been described by Condon and Ogston who also devised the first tool for its analysis (Condon and Ogston, 1966; Ramseyer, 2020a). In the past decade, nonverbal synchrony has received growing attention in multiple areas, because of its simple application based on easily available algorithms from computer-vision (Delaherche et al., 2012). In the domain of psychotherapy, nonverbal synchrony provided valuable information regarding the patient-therapist relationship and their alliance in sessions (Ramseyer and Tschacher, 2011; Altmann et al., 2020; Cohen et al., 2021). Furthermore, it predicted the outcome of psychotherapy and showed relevant associations with relationship and alliance (Ramseyer and Tschacher, 2011, 2014; Prinz et al., 2021; Zimmermann et al., 2021). Whether it is also beneficial for the individual themselves, has recently been questioned (Galbusera et al., 2019).

An objective method to measure the extent of movement-based synchronization is the motion energy analysis (MEA) (Ramseyer and Tschacher, 2011; Ramseyer, 2020b) which relies on simple frame-differencing algorithms. By summing the amount of pixel change occurring between subsequent frames of video recordings, a simple approximation of movement can be extracted. This objective measure of movements may be determined within a specified region of interest (ROI) (Ramseyer, 2020b), thus providing time series of movements for different subjects and/or different regions of a subjects' body. If two or more regions have been defined (e.g., one ROI per subject), a statistical measure of nonverbal synchrony can be quantified using cross-correlational measures (Boker et al., 2002; Tschacher et al., 2014; Schoenherr et al., 2021).

So far, the complex network constituted by the relationship between PHI and their partners has rarely been the focus of research in auditory rehabilitation (Hétu et al., 1993; Ekberg et al., 2015; Scarinci et al., 2021; Völter et al., under revision).

Although a high relationship satisfaction in couples with a hearing-impaired partner has been described in general, low satisfaction was associated with attributions of high causality and responsibility. In case of internal, stable and global causal attributions and intentional responsibility (selfishness or blameworthiness), satisfaction in relationship was low. If the partner judged the hearing loss lower than the hearing-impaired person, the relationship was significantly better than in couples where SOs rated the hearing loss more severe than the partner with hearing impairment (Anderson and Noble, 2005). In a study by Govender, spouses of PHI who felt a change after the onset of the hearing loss reported a severe problem in intimacy and withdrawal from their partner (Govender et al., 2014). This underlines the observation of Knussen et al. (2004) who found that in older PHI (aged 77 years) and their younger SOs (children, aged 45), a poorer relationship led to higher hearing hassles (Knussen et al., 2004).

Generally speaking, understanding the etiology of the hearing loss and being able to express the values and beliefs, like gratitude, humor and optimism, both seem to be mandatory for good interpersonal relations. However, the balance between providing and receiving support on the one hand and allowing and desiring autonomy on the other hand might be difficult (Yorgason et al., 2007). Quite often, the hearing partner takes on a role as buffer, interpreter, mediator, or advocate (Hallberg and Barrenäs, 1993; Hallberg, 1999; Morgan-Jones, 2001). However, these efforts can also lead to an unintended exclusion or an unwanted attention of the PHI and there is a great uncertainty in PHI and SOs over how to involve SOs in the rehabilitation setting of PHI (Scarinci et al., 2021).

Research on the spouses of PHI is still limited to a small number of qualitative studies or small cross-sectional studies (West, 2021). A more profound insight into the relationship dynamics of couple with a hearing-impaired person could ultimately lead to more appropriate psychotherapeutic interventions, which might improve the everyday interaction between PHI and SOs. Taking this potential as a starting point, the aim of our study was (1) to measure nonverbal synchrony in dyads with hearing impairment, (2) to assess and describe potential modifiable factors, and (3) to evaluate the impact of nonverbal synchrony on the interpersonal relationship, the QoL in PHI, and on the burden in SOs.

## Materials and methods

### Participants

Thirty-nine adults (mean age = 63.4 years, SD = 9.66 years) with severe postlingual hearing impairment (herein referred to as PHI) and their significant others (referred to as SOs; mean age = 55.2 years, SD = 15.6 years) participated in the study. Exclusion criteria were severe mental or neurocognitive disorders and limited German language skills. Twenty-nine dyads were spouses with a mean age in PHI of 62.3 years (SD = 9.50; female = 14, male = 15) and in SOs of 62.4 years (SD = 9.14; female = 15, male = 14). All spouses were male/female dyads. The other 10 were dyads of parent (mean age = 66.5 years, SD = 9.97; female = 4, male = 6) and child (mean age = 34.0 years, SD = 10.1; female = 6, male = 4). Degree of hearing impairment was classified according to WHO (average dB of pure tone audiogram at 500, 1000, 2000, and 4000 Hz in the better hearing ear) as follows: WHO 0: <20 dB, WHO 1: 26–40 dB, WHO 2: 41–60 dB, WHO 3: 61–80 dB, WHO 4: > 80 dB.

Twenty-eight PHI had a moderate to severe hearing loss on both sides, 4 PHI had mild hearing loss on the better hearing ear and 7 PHI had single-sided deafness. Thirty-six of the SOs did not report any hearing difficulties, 1 SO had mild and 2 had severe hearing loss.

One out of 40 dyads had to be excluded for the analysis of the nonverbal interaction, and 2 additionally for the analysis of the verbal interaction due to technical errors during recording. The data and measures presented in the study are complete in terms of what was collected for this study. The data supporting the conclusions of this article will be made available by the authors, on reasonable request without undue reservation.

### Setting and video recording

Dyads sitting next to each other in a V-angle in front of a static white background were asked to talk about the organization of an imaginary party that they are going to organize together. Interactions with a duration of 10-min were recorded by a HD camera (Portable Video Lab) using the software VideoSyncProStudio (Mangold^®^).

### Assessment of synchrony

**Figure 1:**
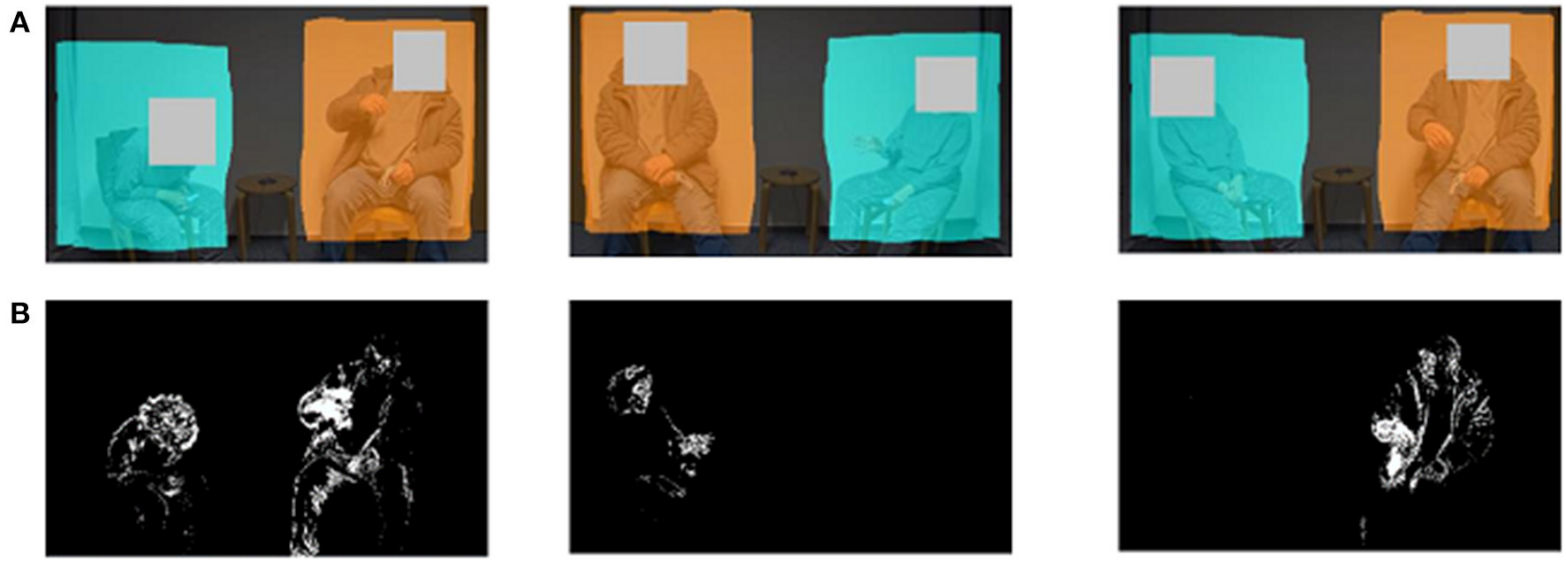
Line **(A)** Original video with regions of interest (ROI) of PHI (left) and SO (right); Line **(B)** Pixels indicate where movements have taken place. PHI, people with hearing impairment; SO, significant other.

**Figure 2:**
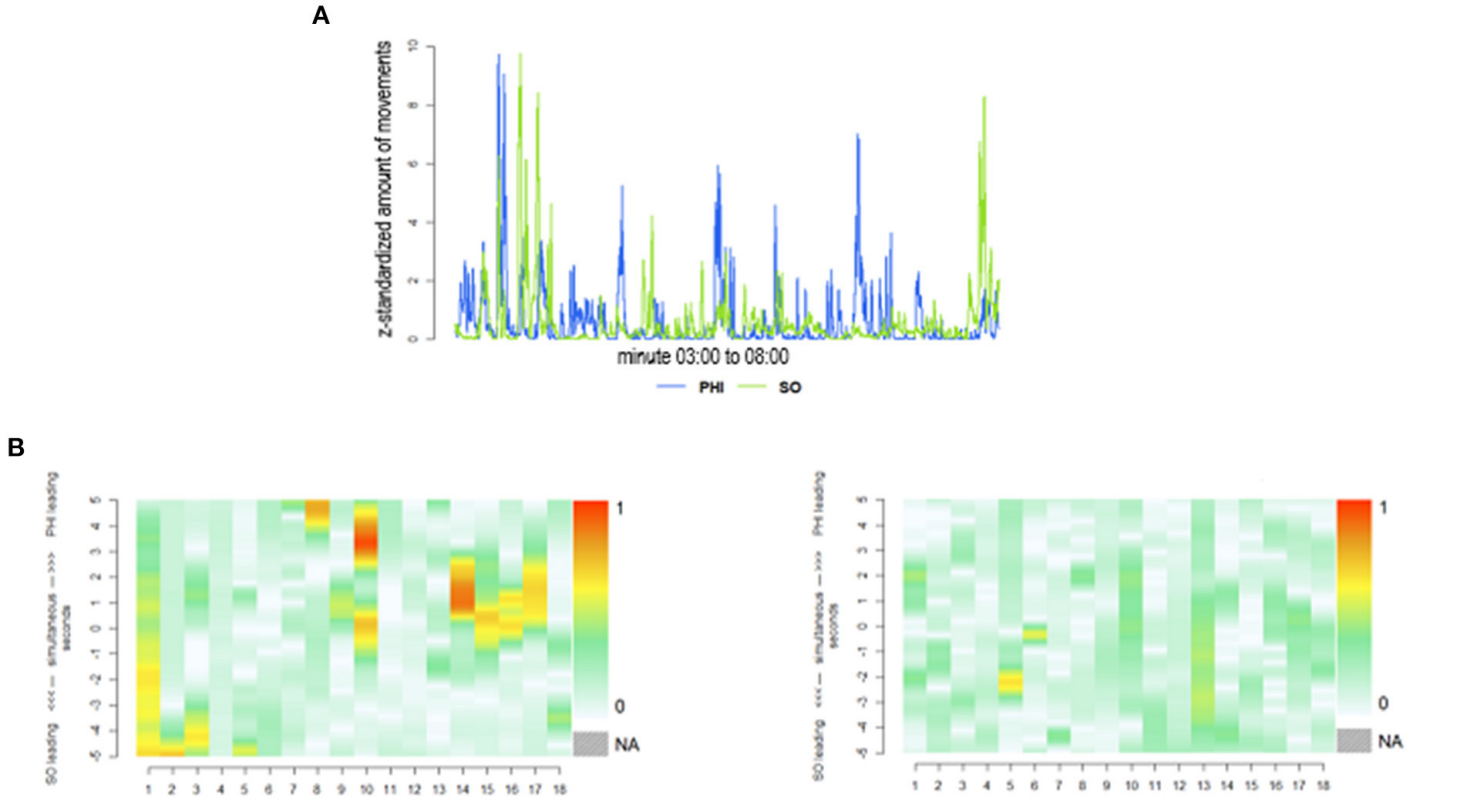
**(A)** Movements of PHI in blue and of SO in green. PHI, people with hearing impairment; SO, significant other. **(B)** Synchronization over time. Examples of dyads with high synchrony, and of dyads with low synchrony. x-axis = time segment of 30 s, y-axis = lags; 0 = synchronic movement, 0–5 = PHI leading, 0 to −5 = SO leading. The more the color turns into orange, the higher the correlation. PHI, people with hearing impairment, SO, significant other.

In line with previous analyses of nonverbal synchrony, videos of the interactions were submitted to an automatized objective quantification of movement using MEA [MEA 4.10 (Ramseyer, 2020b)]. MEA is based on frame-differencing and provides simple time-series of movement dynamics for specific pre-defined regions in recorded interactions. In this study, areas of the head and the upper body were defined as regions of interest (ROI) (Figure 1) and frame-differences were first computed by MEA, and then analyzed in R (R version 4.0.3) using the package rMEA (Kleinbub and Ramseyer, 2020). In line with previous studies using MEA and rMEA, we calculated windowed cross-correlations with suitable parameters for the kind of social interaction: lagged cross-correlations were calculated in segments of 30 s (winSec = 30) with a maximum lag of ±5 s (lagSec = 5), and without overlap (incSec = 30; Figures 2A,B). These parameters and further steps regarding MEA and synchrony are highly similar or identical to two other studies using a comparable paradigm for social interaction (Nelson et al., 2014; Tschacher et al., 2014). Apart from the general strength of synchrony for each dyad (general synchronization), we also differentiated whether PHI or SOs were leading movements, i.e., who tended to move first in the interactions or whether movements were completely synchronic. General synchronization means the absolute mean cross-correlation, which is based on the average of all available lags (lagSec = 5; i.e., lags of ±5 s). Synchronic movements designate the amount of coordination occurring at the exact same time, without any delay. These are the cross-correlations with a lag of 0 s. Apart from these parameters of synchrony, we further established the strength of synchrony by comparing real synchrony with pseudosynchrony (Ramseyer and Tschacher, 2010; Moulder et al., 2018) generated *N* = 1,000 pseudo dyads by between-subject shuffling of all available time series (procedure *shuffle* in rMEA; size = 1,000).

### Assessment of verbal content

Verbal interaction was studied with regard to the total amount of speaking, the amount of speaking in PHI and SOs, the frequency of simultaneous speaking and the number of repetitions and queries by PHI and SOs. The Interact (Mangold^®^) software was used.

### Questionnaires

The following self-report questionnaires were used in order to study health-related quality of life (HRQoL), the burden that the hearing impairment posed on SOs and the quality of the interpersonal relationship:

The ***Nijmegen Cochlear Implant Questionnaire*** (Hinderink et al., 2000) which assesses HRQoL was filled out in the original version by PHI and in a slightly adapted version (Völter et al., under revision) by SOs. The questionnaire consists of 60 questions on a 5-point scale, which can be divided into 6 subscales (basic and advanced sound perception, speech production, self-esteem, activity limitations, social interactions). A lower score means more restrictions in everyday life.

The ***Dyadic Coping Inventory—DCI*** (Bodenmann, 2008) which measures how dyads deal with stress consists of 35 questions on a 5-level scale from very rarely to very often. A higher value indicates better coping strategies. This questionnaire was only used for the subgroup of spouses.

The ***Symptom Checklist short version-9 (SCL-K-*9*)*** (Prinz et al., 2008) was used to assess general signs of psychopathology. Nine items based on 5-point-scales indicate how often the subject felt comfortable or uncomfortable in different situations of psychological distress within the last 7 days. The higher the score, the more distress was found.

The ***Inclusion of Other in the Self Scale (IOS-scale)*** (Aron et al., 1992) measures the closeness experienced in a relationship. Participants are asked to describe the relationship by pointing to a pair of circles which are more or less overlapping, and which are numbered from 1 to 7. Number 7 means the closest relationship. Better quality of relationship is associated with higher closeness (Branand et al., 2019).

The ***SOS-Hear*** Questionnaire first published by Scarinci et al. (2009) is a 5 point-scaled questionnaire for SOs which assesses the burden hearing impairment of PHI poses on the close partner (third-party disability); it was translated into German by Völter et al. (under revision).

### Statistical analysis

Statistical analyses were conducted using the Jamovi (Version 1.8) opensource software. First, descriptive statistics for nonverbal synchrony, verbal aspects, and questionnaires were explored using measures of central tendencies. Impact of gender, degree, and duration of hearing impairment on nonverbal synchrony and verbal interaction were assessed using *t*-tests and one-way ANOVA according to Welch, because of the non-normally distributed values of our measures of synchrony. Associations between age, questionnaires, nonverbal, and verbal interaction were evaluated using Spearman correlation analyses. Scores of nonverbal synchrony and of the questionnaires answered by the spouses and the parent-child dyads were compared with *t*-tests. Significance level was set to *p* < 0.05. Given the explorative character of this study, no correction for multiple testing (e.g., Bonferroni) was applied. Based on experimental data using the same interaction-task in healthy students (Nelson et al., 2014; Tschacher et al., 2014), given a power of 1-*b* = 0.95 and using the previously reported effect-size of Cohen's *d* = 1.11, a sample-size of *N* = 23 for the comparison of synchrony with pseudosynchrony would have been required. The required sample-size is markedly higher when considering the lower effect-size of Cohen's *d* = 0.5, as reported in Nelson et al. (2014), namely *N* = 105. Irrespective of these sample-size considerations, we aimed for a reasonably sized sample with sufficient homogeneity regarding socio-demographic characteristics.

## Results

### Nonverbal synchrony

In this study sample (*N* = 39), nonverbal synchrony was significantly different from pseudosynchrony with an effect size of Cohen's *d* = 0.42 [*t*_(43.4)_ = 2.41; *p* = 0.02]. Table 1 shows means and standard deviations for leading and general synchrony. There was no significant difference between spouses and parent-child dyads (*p* > 0.05) in nonverbal synchrony (general synchrony, PHI leading, SO leading, synchronic movements) and with regard to the self-report questionnaires (*p* > 0.05).

**Table 1:** Results of MEA (motion energy analysis).

	***N***	**Mean**	**Median**	**SD**	**Min**	**Max**
General synchronization	39	0.169	0.165	0.0214	0.138	0.213
PHI leading	39	0.167	0.164	0.0307	0.116	0.254
SO leading	39	0.170	0.168	0.0263	0.126	0.245
Synchronic movements	39	0.183	0.171	0.0458	0.0965	0.286

**Gender** of PHI had a significant influence on general synchrony [*t*_(37)_ = 2.33; *p* = 0.025], especially on the SOs' leading role [*t*_(37)_ = 2.51; *p* = 0.017]: in case of male PHI, SOs took more often the leading position than in female PHI (see Table 2 details).

**Table 2:** Nonverbal synchrony, gender, degree, and duration of hearing impairment.

		**General synchronization**			**PHI leading**			**SO leading**			**Synchronic movements**		
	***N***	**Mean**	**SD**	***p***	**Mean**	**SD**	***p***	**Mean**	**SD**	***p***	**Mean**	**SD**	***p***
**Gender (PHI)**													
Male	21	0.176	0.021		0.172	0.035		0.179	0.025		0.193	0.048	
Female	18	0.160	0.019	**0.025**	0.161	0.025	0.295	0.159	0.023	**0.017**	0.173	0.042	0.187
**Degree of hearing impairment in PHI**													
WHO 0	7	0.173	0.026		0.180	0.032		0.166	0.041		0.185	0.067	
WHO 1	4	0.154	0.018		0.155	0.024		0.153	0.025		0.172	0.024	
WHO 2	3	0.173	0.012		0.145	0.027		0.200	0.010		0.210	0.006	
WHO 3	8	0.159	0.013		0.157	0.022		0.162	0.011		0.164	0.029	
WHO 4	17	0.174	0.023	0.292	0.173	0.034	0.386	0.174	0.023	**0.007**	0.190	0.049	**0.010**
**Duration of hearing impairment in PHI**													
<2	4	0.150	0.009		0.161	0.019		0.139	0.012		0.159	0.044	
2–5	4	0.180	0.024		0.166	0.018		0.195	0.039		0.202	0.052	
6–10	4	0.164	0.022		0.160	0.012		0.167	0.036		0.169	0.033	
11–20	4	0.189	0.019		0.191	0.054		0.187	0.025		0.232	0.045	
>20	23	0.167	0.020	0.053	0.165	0.032	0.869	0.169	0.019	**0.031**	0.179	0.044	0.310

Significance level was set at p < 0.05 and is written in bold. PHI, people with hearing impairment; SO, significant others.

**Age** of PHI and of SOs was not associated with synchrony (*p* = 0.087–0.997), but the **age gap** between PHI and SOs negatively correlated with synchronic movements (*rho* = −0.320; *p* = 0.047). The smaller the age gap was, the higher synchrony was (see Table 3). Regarding only dyads of spouses, the age gap correlated with synchronic movements (*rho* = −0.453; *p* = 0.014) and the SOs leading role (*rho* = −0.488; *p* = 0.007).

**Table 3:** Correlation nonverbal synchrony with age, age gap, and questionnaires (*N* = 39).

		**General synchronization**	**PHI leading**	**SO leading**	**Synchronic movements**
Age (PHI)	*rho*	0.057	0.115	−0.064	0.001
	*p*	0.730	0.487	0.698	0.997
Age (SO)	*rho*	−0.115	0.083	−0.278	−0.104
	*p*	0.484	0.615	0.087	0.530
Age gap	*rho*	−0.228	−0.221	−0.141	−0.320
	*p*	0.162	0.177	0.390	**0.047**
Nijmegen (PHI)	*rho*	−0.038	−0.138	0.018	−0.062
	*p*	0.820	0.404	0.916	0.708
Nijmegen (SO)	*rho*	−0.148	−0.110	−0.177	−0.047
	*p*	0.368	0.507	0.280	0.776
DCI (PHI)	*rho*	0.020	0.090	−0.125	0.044
	*p*	0.919	0.644	0.518	0.821
DCI (SO)	*rho*	0.054	0.018	−0.066	0.200
	*p*	0.781	0.925	0.734	0.299
SCL-K-9 (PHI)	*rho*	0.162	0.169	0.020	0.086
	*p*	0.325	0.304	0.901	0.604
SCL-K-9 (SO)	*rho*	−0.051	−0.112	0.024	0.076
	*p*	0.758	0.498	0.886	0.646
IOS (PHI)	*rho*	−0.246	0.137	−0.521	−0.019
	*p*	0.131	0.405	**<0.001**	0.907
IOS (SO)	*rho*	0.068	0.387	−0.295	0.006
	*p*	0.679	**0.015**	0.069	0.971
SOS-HEAR	*rho*	−0.041	−0.041	0.027	−0.028
	*p*	0.805	0.806	0.868	0.866

Correlation of DCI and nonverbal synchrony of spouses only (N = 29). Significance level was set at p < 0.05 and is written in bold.

**Degree of hearing impairment** had an influence on the SOs leading role [*F*_(4/9.52)_ = 6.77; *p* = 0.007]. Subjects with a moderate hearing impairment (WHO group 2) experienced more leading by the SOs than those with a more severe hearing impairment [WHO group 3; *t*_(4.06)_ = 5.483; *p* = 0.024]. Degree of hearing impairment was also associated with synchronic movements [*F*_(4/12.25)_ = 5.43; *p* = 0.010], and there was a difference between WHO groups 2 and 3 [*t*_(8.37)_ = 4.144; *p* = 0.018].

**Duration of hearing impairment** had an impact on the SOs leading role [*F*_(4/7.24)_ = 4.946; *p* = 0.031] and an impact at trend-level on general synchrony [*F*_(4/7.88)_ = 3.775; *p* = 0.053], but not on the amount of synchronic movements [*F*_(4/7.48)_ = 1.442; *p* = 0.310] or the PHIs' leading role [*F*_(4/8.41)_ = 0.302; *p* = 0.869]. More specifically, in hearing-impaired subjects who have suffered from hearing loss for <2 years, SOs had a less strong leading position. PHI who had had hearing loss for <2 years differed in the SOs' leading role from the PHI with a hearing loss for 2.5- 5 years [*t*_(34)_ = −3.38; *p* = 0.015] and the PHI with a hearing loss for 11–20 years [*t*_(34)_ = −2.922; *p* = 0.045]: Their leading was lower than the SO leading of the other groups.

**Health related quality of life (HRQoL)** assessed by the Nijmegen Questionnaire was rated quite similar in PHI and SOs [*t*_(38)_ = 0.866; *p* = 0.392, Table 4]. HRQoL was not associated with synchrony (*p* > 0.05, Table 3), neither in the total score nor in the subscores (*p* > 0.05).

**Table 4:** Results of the different questionnaires applied.

	***N***	**Mean**	**Median**	**SD**	**Min**.	**Max**.
Nijmegen (PHI)	39	59.60	60.60	14.70	22.40	84.30
Nijmegen (SO)	39	57.90	58.50	15.50	17.10	88.80
DCI (PHI)	29	125.00	122.00	14.30	100.00	158.00
DCI (SO)	29	122.00	124.00	15.00	85.00	153.00
SCL-K-9 (PHI)	39	0.71	0.56	0.55	0.00	2.67
SCL-K-9 (SO)	39	0.61	0.44	0.48	0.00	2.11
IOS (PHI)	39	6.21	6.00	0.92	4.00	7.00
IOS (SO)	39	6.03	6.00	1.09	4.00	7.00
SOS-HEAR	39	1.11	0.89	0.75	0.11	3.11

DCI from spouses only.

**Coping mechanisms** (only available for spouses) were also quite similar between PHI and the SOs [*t*_(28)_ = 1.18; *p* = 0.249]. On the Dyadic Coping Inventory (DCI), PHI scored 125 (*SD* = 14.3) and SOs 122 (*SD* = 15.0) out of 175 points, both indicating an average coping mechanism according to Bodenmann (2008). No significant correlation was found between the total coping score or any subscore and synchrony (*p* > 0.05).

**Psychopathology** measured by SCL-K-9 was rated slightly higher by PHI (*M* = 0.71; *SD* = 0.55) than by SOs (*M* = 0.61; *SD* = 0.48), although not significant [*t*_(38)_ = 1.016; *p* = 0.316]. No correlation was found between nonverbal synchrony and SCL-K-9 in PHI and in SOs (*p* > 0.05).

**Burden** in SOs measured by the SOS-Hear was rated *M* = 1.11 on average (*SD* = 0.75). There was no correlation between SOS-Hear and nonverbal synchrony (*p* > 0.05).

**Closeness** assessed by the inclusion of other in the self scale (IOS) was on average quite similar in PHI (*M* = 6.21, *SD* = 0.92) and SOs [*M* = 6.03, *SD* = 1.09; *t*_(38)_ = 1.069; *p* = 0.292]. A significant negative correlation was found between IOS of PHI and the SOs' leading role (*rho* = −0.521; *p* = < 0.001), and a significant positive correlation between IOS of SOs and PHI's leading role (*rho* = 0.387; *p* = 0.015). When PHI reported a close relationship, SOs were less leading; if SOs reported about a close relationship, PHI lead more.

### Verbal interaction

Verbal interaction took place in 74% of the recorded time. In 41%, the person with hearing impairment was talking; in 33% the SO was talking. Both spoke at the same time 3% of the time. PHI had a significant higher percentage of speaking than the SOs [*t*_(36)_ = 2.84; *p* = 0.007]. SOs repeated utterances 2.95 times and PHI 0.29 times on average. PHI posed questions 1.95 times and SOs 0.297 times on average. **Nonverbal synchrony** (general synchrony, SO leading, PHI leading, synchronic movements) did not correlate with percentage of speaking by the PHI, the SOs or simultaneous speaking of both and with the repetition of utterances and posed questions (*p* > 0.05). Only in the subgroup of PHI with a severe hearing loss (*N* = 24), the percentage of speaking by PHI negatively correlated with PHI leading (*rho* = −0.453; *p* = 0.027).

**Age of PHI** and **of SOs**, **age gap, gender** and **duration of hearing impairment** did not show a correlation with verbal interaction (*p* > 0.05). There was also no correlation between the **degree of hearing impairment** with the percentage of verbal interaction by PHI and SOs (*p* > 0.05). However, the degree of hearing impairment positively correlated with the frequency of repetitions by SOs (*rho* = 0.407; *p* = 0.013) and with the queries by PHI (*rho* = 0.331; *p* = 0.045), but not with the queries by SOs and repetitions of the PHI (*p* > 0.05; Table 5).

**Table 5:** Correlation of verbal interaction with demographic data and questionnaires of spouses (*N* = 37).

		**Queries (PHI)**	**Queries (SO)**	**Repetitions (PHI)**	**Repetitions (SO)**
Age (PHI)	*rho*	0.089	−0.212	−0.245	0.132
	*p*	0.601	0.208	0.144	0.435
Age (SO)	*rho*	0.186	0.142	0.114	0.056
	*p*	0.271	0.403	0.503	0.741
Age gap	*rho*	0.015	−0.111	−0.119	0.198
	*p*	0.928	0.514	0.484	0.241
Degree of hearing impairment	*rho*	0.331	0.094	0.068	0.407
	*p*	**0.045**	0.580	0.691	**0.013**
Duration of hearing impairment	*rho*	0.064	0.019	0.001	0.183
	*p*	0.705	0.910	0.993	0.279
Nijmegen (PHI)	*rho*	−0.560	0.033	0.037	−0.589
	*p*	**<** **0.001**	0.847	0.827	**<** **0.001**
Nijmegen (SO)	*rho*	−0.479	−0.056	−0.063	−0.605
	*p*	**0.003**	0.743	0.711	**<** **0.001**
DCI (PHI)	*rho*	−0.174	−0.453	−0.468	−0.177
	*p*	0.387	**0.018**	**0.014**	0.378
DCI (SO)	*rho*	−0.493	−0.185	−0.228	−0.475
	*p*	**0.009**	0.355	0.253	**0.012**
SCL-K-9 (PHI)	*rho*	0.347	−0.070	−0.030	0.214
	*p*	**0.035**	0.678	0.859	0.203
SCL-K-9 (SO)	*rho*	0.162	0.111	0.106	0.258
	*p*	0.337	0.514	0.534	0.123
IOS (PHI)	*rho*	−0.198	−0.073	−0.115	−0.065
	*p*	0.241	0.667	0.497	0.701
IOS (SO)	*rho*	0.037	0.125	0.085	−0.046
	*p*	0.826	0.462	0.616	0.787
SOS-HEAR	*rho*	0.403	0.013	0.029	0.600
	*p*	**0.013**	0.940	0.866	**<0.001**

Due to technical problems during video recording, the verbal patterns of 2 spouses could not be evaluated. Correlations of DCI of spouses only (N = 27). Significance level was set at p < 0.05 and is written in bold.

No correlation was found between the percentage of speaking by PHI and by SOs or with simultaneous speaking and **Nijmegen**, **SCL-K-9**, or the **SOS-Hear** questionnaires (each *p* > 0.05). Coping mechanisms of SOs assessed by **DCI** in spouses positively correlated with the percentage of speaking by SOs (*rho* = 0.418; *p* = 0.030). Furthermore, there was a correlation between **IOS** of PHI and simultaneous speaking (*rho* = 0.386; *p* = 0.018) and the total percentage of speaking (*rho* = 0.328; *p* = 0.048).

However, the number of queries by the PHI negatively correlated with HRQoL assessed by the **Nijmegen** score in PHI (*rho* = −0.560; *p* < 0.001) and SOs (*rho* = −0.479; *p* = 0.003). Repetitions by SOs showed a negative correlation with the HRQoL assessed by the Nijmegen Questionnaire in PHI (*rho* = −0.589; *p* < 0.001) and SOs (*rho* = −0.605; *p* < 0.001). The **DCI score** of PHI correlated with repetitions of the PHI (*rho* = −0.468; *p* = 0.014) and the queries by the SOs (*rho* = −0.453; *p* = 0.018). The DCI score of SOs showed a correlation with the number of queries of the PHI (*rho* = −0.493; *p* = 0.009) and with the repetitions of SOs (*rho* = −0.475; *p* = 0.012). Coping strategies applied by the SOs were the better, the less questions were posed by the PHI and the less repetitions were made by the SOs. **Psychopathology** of the SOs assessed by SCL-K-9 had no impact on the number of queries and repetitions by the PHI or SOs (*p* > 0.05). Mood of PHI correlated with the posed queries by PHI (*rho* = 0.347; *p* = 0.035). Closeness felt by PHI and SOs was not associated with queries and repetitions by PHI and SOs (*p* > 0.05). **Burden** experienced by SOs assessed by the SOS-Hear was positively associated with queries posed by PHI (*rho* = 0.403; *p* = 0.013) and repetitions by SOs (*rho* = 0.600; *p* < 0.001).

### Questionnaires

There was a negative correlation between the **Nijmegen** and the **SCL-K-9** in PHI (*rho* = −0.420; *p* = 0.008, Table 6). The lower the restrictions were in everyday life that PHI had to complain about, the less symptomatology was reported by the PHI. **SOS-Hear** negatively correlated with the **Nijmegen** score of PHI (*rho* = −0.363; *p* = 0.023) and SOs (*rho* = −0.712; *p* < 0.001). The better the HRQoL as judged by PHI and SOs was, the less third-party disability was described by SOs. The **Nijmegen score** of SOs significantly correlated with PHI's **DCI** (*rho* = 0.397; *p* = 0.033) and SOs' DCI (*rho* = 0.526; *p* = 0.003). The better the handling of stress by PHI and SOs was, the less SOs perceived the restrictions caused by their partners' hearing impairment in everyday life. There was a negative correlation between SOS-Hear and SOs' DCI (*rho* = −0.429; *p* = 0.020). The better the handling of stress was in SOs, the less pronounced the third-party disability was. DCI of PHI also correlated with DCI of SOs (*rho* = 0.508; *p* = 0.005). There was a negative correlation between **DCI** of SOs and **SCL-K-9** of SOs (*rho* = −0.443; *p* = 0.016). **SCL-K-9** of PHI correlated with the **IOS** score of PHI (*rho* = −0.365; *p* = 0.022). The closer the relationship was, the less symptomatology was reported. The IOS score of PHI correlated with the IOS of SOs (*rho* = 0.329; *p* = 0.041). A correlation was found between **SOS-Hear** and **SCL-K-9** in SOs (*rho* = 0.439; *p* = 0.005) and SOS-Hear and the **IOS** score in SOs (*rho* = −0.392; *p* = 0.014). The worse the mood of SOs, the higher the third-party disability. The closer SOs assessed the relationship, the less third-party disability was claimed.

**Table 6:** Correlation between the questionnaires of spouses (*N* = 39).

		**Nijmegen**	**Nijmegen**	**DCI**	**DCI**	**SCL-K-9**	**SCL-K-9**	**IOS**	**IOS**
		**(PHI)**	**(SO)**	**(PHI)**	**(SO)**	**(PHI)**	**(SO)**	**(PHI)**	**(SO)**
Nijmegen (PHI)	*rho*	–							
	*p*	–							
Nijmegen (SO)	*rho*	0.619							
	*p*	**<0.001**							
DCI (PHI)	*rho*	0.233	0.397	–					
	*p*	0.224	**0.033**	–					
DCI (SO)	*rho*	0.232	0.526	0.508	–				
	*p*	0.225	**0.003**	**0.005**	–				
SCL-K-9 (PHI)	*rho*	−0.420	−0.109	−0.241	−0.072	–			
	*p*	**0.008**	0.510	0.209	0.712	–			
SCL-K-9 (SO)	*rho*	−0.071	−0.293	−0.123	−0.443	0.134	–		
	*p*	0.668	0.070	0.526	**0.016**	0.416	–		
IOS (PHI)	*rho*	−0.070	−0.070	0.198	0.133	−0.365	−0.135	–	
	*p*	0.674	0.671	0.304	0.492	**0.022**	0.411	–	
IOS (SO)	*rho*	0.017	0.156	0.114	0.188	0.041	−0.182	0.329	–
	*p*	0.919	0.342	0.555	0.328	0.804	0.266	**0.041**	–
SOS-HEAR	*rho*	−0.363	−0.712	−0.181	−0.429	−0.045	0.439	0.020	−0.392
	*p*	**0.023**	**<0.001**	0.346	**0.020**	0.784	**0.005**	0.901	**0.014**

Significance level was set at p < 0.05 and is written in bold. Correlations of DCI of spouses only (N = 29).

## Discussion

To our knowledge, this is the first empirical exploration of nonverbal synchrony in PHI. Our data show that the phenomenon of synchrony is present in dyads with one hearing-impaired subject and their SO. The degree of synchrony (average cross-correlation) in dyads with one hearing-impaired partner is comparable to interactions with the same task performed by healthy students (Nelson et al., 2014; Tschacher et al., 2014), and it is higher than generally reported in psychotherapy dyads (Ramseyer and Tschacher, 2011; Cohen et al., 2021). In terms of the effect-size derived from the comparison with pseudosynchrony, we found a lower or similar effect (Cohen's *d* = 0.42) in dyads with an hearing-impaired subject than the one reported in student dyads who had not known each other before the interaction (*d* = 1.11; Tschacher et al., 2014), and in students interacting in a setting with further experimental factors (*d* = 0.54; Nelson et al., 2014). In psychotherapy dyads, using the same methodology, the effect size was higher (*d* = 0.60; Ramseyer and Tschacher, 2011). Our interpretation of this finding is that many hearing-impaired people withdraw from social activities and thus have less social contacts. The (potential) loneliness associated with this state of living has been associated with decreased interpersonal synchrony (Saporta et al., 2022).

The most distinctive findings from the dataset are the differential associations between demographic factors, questionnaire data and the leading role of SOs: on a general level, more leading by a non-impaired SO was associated with problematic aspects of the duration of hearing impairment (PHI), the age gap, and a lower overlap of self and other (IOS). Gender of PHI had an effect on the nonverbal synchrony as well.

In male PHI the SO took more often the nonverbally leading position than in female PHI. This could reflect that the impact of the hearing impairment is greater on wives of a male PHI (Wallhagen et al., 2004; Anderson and Noble, 2005). In general, gender effects have been found with more synchrony in same-gender-dyads of females than in same-gender-dyads of males and with regard to anti-phase patterning (Fujiwara et al., 2019). Our study supports that even in different gender dyads, gender effects can be observed. Furthermore, a longer hearing impairment leads to more nonverbal leading by SOs than in PHI with very short duration of hearing loss, possible be due to long-standing imbalance in communication.

Stronger leading by SOs was associated with less self-other overlap reported by PHI, i.e., a person with hearing impairment who is nonverbally led by their SO reports lower overlap than a person where leading is less prominent. Although many of the questionnaires did fail to show significant associations with nonverbal synchrony (may be also due to the small sample-size), we think that the distinctive pattern visible in the IOS suggests the following interpretation: PHI who are led by their SOs to a high degree, report less closeness to the SO than PHI experiencing lower levels of being led. We may thus conclude that in PHI, the experience of being led by a SO could be related to a potentially reactive stance of the healthy SO. We use the term reactive because the complications imposed by the hearing loss might lead to frustration in the SO, which in turn may lead to a more dependent role of the PHI. In our small sample reported here, this dependence may be interpreted as a sign of deterioration of the relationship that becomes obvious in lower ratings of closeness in these cases.

In dyads with a small age gap, more synchronic movements appear and especially in spouses with a small age gap, SOs were even more leading than in those with a large age gap. This differential effect of similar age vs. larger age-difference could be interpreted as a saliency-effect (Humphreys and Sui, 2015): a SO with a spouse of similar age may cope with the impairments imposed by hearing loss in a different way, specifically in comparison with a SO who has an older partner with impairments that may make the hearing loss stand out in a less salient way and thus impose less of an experience of burden on them.

The distinction between leading and following has not been fully explored in most previous reports based on the MEA-methodology, possibly also because of an inherent difficulty assigning specific roles in, e.g., student interactions. However, one recent study from the domain of psychotherapy interactions found that therapist's following in sessions 3 and 8 of psychotherapy (i.e., the beginning phase of therapy) predicted higher depressive symptoms and more interpersonal problems reported by patients at the end of therapy (Altmann et al., 2020). Another study based on MEA found that patients diagnosed with borderline personality disorder (BPD) refrained from imitating their interview partner, specifically after the administration of intranasal oxytocin, while healthy controls did not show such a specific effect on their imitation pattern (Ramseyer et al., 2020).

Generally speaking, a wide range of associations was found between nonverbal synchrony and different aspects of psychotherapy. In a number of more recent studies, synchrony was not unequivocally a sign of positive development or of good relationships, as higher levels of synchrony have been observed after so-called ruptures in therapeutic relationships (Deres-Cohen et al., 2021), and in the setting of coaching, high levels of nonverbal synchrony have been considered to be corrective efforts initiated by the coach, aimed at reestablishing or improving the coaching relationship (Erdös and Ramseyer, 2021). This initially counterintuitive association between nonverbal synchrony and deterioration has been previously reported in marital interaction (Levenson and Gottman, 1983); in a therapeutic or coaching context, high levels of synchrony may be conceptualized as indicators of synchrony serving the role of a social glue (Lakin et al., 2003). In fact, similar to the metaphor of social glue, nonverbal synchrony has also been found with a possible aim to create a good climate: A recent study in the medical context suggested that nonverbal synchrony was higher in racially-discordant dyads (vs. concordant dyads), and that patient's positive affect and patient's positive rapport were positively associated with synchrony in discordant dyads, while no significant association was found in racially-concordant patient-physician dyads (Hamel et al., 2022).

### Clinical implications

Due to the explorative nature of this study, caution needs to be applied regarding potential clinical implications of the findings reported above: first of all, we think that similar to the psychotherapy setting, nonverbal synchrony embodies aspects of the relationship between PHI and their SOs. These aspects were assessed by the relatively easy and unobtrusive way of MEA and simple video-recordings of scripted interactions in order to find a more nuanced understanding of the impact of hearing loss on both individuals in a relationship.

One of the main findings implies that nonverbal dominance (i.e., who is leading the interaction on the level of body-movement) was associated with how close PHI and SOs felt to their respective partners. It could be argued that raising SOs' awareness of leading or not-leading interactions at the level of body movement could provide a simple clue regarding the relational closeness between the partners. Furthermore, our finding of a lower effect size than in dyads of students suggests that a potential social withdrawal caused by the hearing impairment may influence nonverbal synchrony. Therefore, the social setting (activity and participation) should be considered in future assessments. Both loneliness (Saporta et al., 2022) and social anxiety (Asher et al., 2020) appear to influence interpersonal coordination.

Interventions targeting the use or misuse of dominance in everyday interactions could thus lessen the burden on both partners and at the same time provide a simple way of assessing potentially problematic dynamics in this domain. The 10-min interaction we used in this study presents little burden on both participants, but it provides a first glimpse of the dynamics unfolding between PHI and SOs. Future studies should (a) broaden the assessment of relationship parameters, and (b) experimentally assess whether raising SOs' awareness of nonverbal dominance could have an effect on the HRQoL in PHI and their SOs. Furthermore, parameters which may also influence interpersonal relationships, such as socioeconomic status, cultural background, other chronic diseases or social inclusion, were not included in the study and should be studied in detail in future projects.

Gender may lead to different experiences in dealing with chronic diseases, such as hearing impairment. In general, men are more likely to rely on a spouse for emotional support, and caregiving women who find more social support outside the marital relationship have more resources to cope with the spouses' hearing impairment and can provide more emotional support to their dyad (Thomeer et al., 2015; Behler et al., 2018). Thus, health care providers need to be aware of the implications for husbands when treating women with hearing impairment (West, 2021).

Nowadays, various surgical and non-surgical options are available to treat a chronic hearing loss (Löhler et al., 2019). In case of a severe- to profound hearing loss with little impact of conventional hearing aids, an electronic inner ear prosthetic device called cochlear implant (CI) is the method of choice (Dazert et al., 2020). CIs directly stimulate the auditory nerve, which results in significant improvements in speech understanding within the first 6 months (Lenarz, 2018; Boisvert et al., 2020; Carlson, 2020). Furthermore, positive outcomes of auditory rehabilitation with regard to HRQoL, psychosocial comorbidities, and cognitive functions have been reported (Völter et al., 2021). Therefore, it might be interesting to evaluate whether an improvement in auditory functions by auditory rehabilitation *via* cochlear implantation might have an impact on interpersonal synchrony in the long-term follow-up.

### Limitations

As declared in the methods section, this study was not solely guided by a specific hypothesis-driven approach to the phenomenon of nonverbal synchrony: considering the fact that findings reported in the psychotherapy setting are sometimes contradictory, we sought to explore the phenomenon in the hitherto unexplored domain of hearing impairment. Apart from the explorative nature of this study, the sample size appears relatively low, thus caution regarding the transferability of the results should be applied. As the present study employed an observational and cross-sectional design, direct causality inferences could not be made.

### Summary

This study reported nonverbal synchrony in dyads with at least one member suffering from hearing impairment. Main findings suggested that nonverbal synchrony—assessed by quantifying the leading role within dyads—predicted lower experienced closeness between interaction partners. A dyads' way to deal with the difficult situation of impaired communication possibilities was partially embodied by the dynamics of the healthy and hearing-impaired participant during a natural conversation. This finding encourages further use of nonverbal signals in rehabilitation and management of hearing impairment, which could ultimately lead to improvement of communication abilities in both parties.

## Data availability statement

The data supporting the conclusions of this article will be made available by the authors, on reasonable request, without undue reservation.

## Ethics statement

The studies involving human participants were reviewed and approved by Ethics Institution of the Ruhr University of Bochum, Germany (No. 20-6851). The patients/participants provided their written informed consent to participate in this study.

## Author contributions

CV designed the study. SM and KO selected the subjects and collected the data. CV, FR, SM, and KO analyzed and evaluated the data. CV, FR, and KO wrote the manuscript with contributions and critical feedback from all authors. All authors contributed to the article and approved the submitted version.

## Funding

A part of the study was supported by the FoRUM-Program of the Ruhr-University Bochum (F1017-2021). We acknowledge support by the DFG Open Access Publication Funds of the Ruhr-Universität Bochum.

## Conflict of interest

The authors declare that the research was conducted in the absence of any commercial or financial relationships that could be construed as a potential conflict of interest.

## Publisher's note

All claims expressed in this article are solely those of the authors and do not necessarily represent those of their affiliated organizations, or those of the publisher, the editors and the reviewers. Any product that may be evaluated in this article, or claim that may be made by its manufacturer, is not guaranteed or endorsed by the publisher.

## Abbreviations

PHI: people with hearing impairment
SO: significant other.
